# CCR2 and CXCR3 agonistic chemokines are differently expressed and regulated in human alveolar epithelial cells type II

**DOI:** 10.1186/1465-9921-6-75

**Published:** 2005-07-20

**Authors:** Dmitri V Pechkovsky, Torsten Goldmann, Corinna Ludwig, Antje Prasse, Ekkehard Vollmer, Joachim Müller-Quernheim, Gernot Zissel

**Affiliations:** 1 Department of Pneumology, Medical Center, Albert-Ludwigs University, Freiburg, Germany; 2 Research Institute for Pulmonary Diseases and Tuberculosis, Minsk, Belarus; 3 Division of Infectious Diseases, University of British Columbia, Vancouver, British Columbia V5Z 3J5, Canada; 4 Division of Clinical and Experimental Pathology, Research Center Borstel, Borstel, Germany; 5 Department of Thoracic Surgery, Albert-Ludwigs University, Freiburg, Germany; 6 Lungenklinik, Krankenhaus Merheim, Kliniken der Stadt Köln, Köln, Germany

## Abstract

The attraction of leukocytes from circulation to inflamed lungs depends on the activation of both the leukocytes and the resident cells within the lung. In this study we determined gene expression and secretion patterns for monocyte chemoattractant protein-1 (MCP-1/CCL2) and T-cell specific CXCR3 agonistic chemokines (Mig/CXCL9, IP-10/CXCL10, and I-TAC/CXCL11) in TNF-α-, IFN-γ-, and IL-1β-stimulated human alveolar epithelial cells type II (AEC-II). AEC-II constitutively expressed high level of CCL2 mRNA *in vitro *and *in situ *, and released CCL2 protein *in vitro *. Treatment of AEC-II with proinflammatory cytokines up-regulated both CCL2 mRNA expression and release of immunoreactive CCL2, whereas IFN-γ had no effect on CCL2 release. In contrast, CXCR3 agonistic chemokines were not detected in freshly isolated AEC-II or in non-stimulated epithelial like cell line A549. IFN-γ, alone or in combination with IL-1β and TNF-α resulted in an increase in CXCL10, CXCL11, and CXCL9 mRNA expression and generation of CXCL10 protein by AEC-II or A549 cells. CXCL10 gene expression and secretion were induced in dose-dependent manner after cytokine-stimulation of AEC-II with an order of potency IFN-γ>>IL-1β ≥ TNF-α. Additionally, we localized the CCL2 and CXCL10 mRNAs in human lung tissue explants by *in situ *hybridization, and demonstrated the selective effects of cytokines and dexamethasone on CCL2 and CXCL10 expression. These data suggest that the regulation of the CCL2 and CXCL10 expression exhibit significant differences in their mechanisms, and also demonstrate that the alveolar epithelium contributes to the cytokine milieu of the lung, with the ability to respond to locally generated cytokines and to produce potent mediators of the local inflammatory response.

## Background

Many pulmonary disorders are characterized by accumulation and activation of inflammatory cells within the lung, followed by the release of regulatory mediators, resulting in macrophage/lymphocyte alveolitis. Sarcoidosis, tuberculosis, hypersensitivity pneumonitis, eosinophilic pneumonia, and usual interstitial pneumonia represent such lung diseases that have in common the selective recruitment and activation of different types of leukocytes, and therefore, exhibit distinct forms of alveolitis [1-5]. The inflammatory phase of alveolitis is initiated by epithelial and/or endothelial injury involving the structures of the alveolar wall. The alveolar surface area of the lung is covered with a layer of alveolar epithelial cells type I and type II. Type I cells function as a physical barrier, whereas type II cells produce surfactant and act as progenitors to replace injured alveolar epithelial cells type I [6]. Thus, located at the boundary between the alveolar airspace and the interstitium, alveolar epithelial cells type II (AEC-II) are ideally situated to regulate the recruitment and activation of different types of leukocytes through the production of chemokines/cytokines in response to inflammatory stimulation from the alveolar space. Recent studies have suggested that AEC-II secrete a variety of mediators, including proinflammatory cytokines and chemokines important for the recruitment of monocytes / macrophages and T cells into the lung interstitium and alveolar space [7-10].

Although leukocyte recruitment is a complex and multistep process with involvement of different types of cells, cell-surface adhesion molecules, and soluble inflammatory mediators, the prominent role of the attractant molecules such as chemokines has widely been appreciated [11,12]. Chemokines are a superfamily of small, secreted proteins that direct the recruitment of leukocytes to the sites of inflammation. They are classified into four subfamilies on the basis of the primary sequence of the first two of four invariant cysteine residues, and named according to the recommendation for new systematic nomenclature for human chemokines [11]. CC chemokines/CCL attract monocytes, eosinophils, basophils, dendritic and T cells and signal through chemokine receptors CCR1 to CCR10. In contrast to CC chemokines, the CXC chemokines (CXCL) are divided into two classes depending on the presence of the glutamate-leucine-arginine motif (ELR) in the NH_2_-terminal domain. The CXC chemokines signal through the chemokine receptors CXCR1 to CXCR5 (reviewed in [11]). The CC chemokine, monocyte chemoattractant protein-1/CCL2 (CCL2), has been shown *in vitro *and *in vivo *to target preferentially monocytes and memory T cells through the CCR2 [13-16]. Monokine induced by IFN-γ (Mig/CXCL9), IFN-induced protein of 10 kDa (IP-10/CXCL10), and IFN-inducible T-cell α-chemoattractant (I-TAC/CXCL11) are all members of the non-ELR CXCL class and target preferentially memory T cells and natural killer cells through the single and shared receptor CXCR3 [17,18]. Recently, it has been reported that some chemokine receptors are associated with human Th1 or Th2 cells, and therefore the respective agonists can selectively attract the respective Th cell subset into inflammatory sites (reviewed in [12]).

In this context, we hypothesized that AEC-II are an important source of CCL2 and the CXCR3 agonistic chemokines in the lung, and through expression of these mediators involved in the homing of immune effector cells during lung inflammatory processes. As a model we investigated the gene expression and production of chemokines, important for the recruitment of CCR2 and CXCR3 bearing mononuclear leukocytes, by human primary AEC-II and airway epithelial like cell line A549 after exposure of the cells to the proinflammatory cytokines TNF-α, IFN-γ, and IL-1β. A striking result was the difference between spontaneous and cytokine-induced CCL2, CXCL9, CXCL10, and CXCL11 mRNA expression and/or protein production in both human AEC-II and A549 cell cultures. Finally, we provide evidence of selective CCL2 and CXCL10 mRNA expression of human AEC-II *in vivo *.

## Materials and Methods

### Reagents

The following materials were purchased from GIBCO BRL (Paisley, Scotland): PBS, RPMI 1640 medium with 2 mM L-glutamine, FCS, HEPES, TRIZOL Reagent, SuperScript™ RNase H^- ^reverse transcriptase (RT), oligo (dT)_12–18 _primer and agarose; penicillin/streptomycin solution and sodium pyruvate from Biochrom (Berlin, FRG); trypsin/EDTA solution from Boehringer-Mannheim (Mannheim, FRG); collagen R from Serva (Heidelberg, FRG); chloroform and isopropanol from Merck (Darmstadt, FRG); recombinant human IFN-γ (specific activity 3 × 10^7 ^U/mg) and recombinant human IL-1β (specific activity 2 × 10^8 ^U/mg) from Biotrend (Cologne, FRG); recombinant human TNF-α was a courtesy of Dr. E. Schlick (Knoll AG, Ludwigshafen, FRG); dexamethasone from Sigma (St. Louis, MO); 100 mm plastic dishes, 75 cm^2 ^tissue culture flask and 24-well cell culture plates from NUNC (Wiesbaden, FRG). All reagents used were of the highest available grade and were dissolved in pyrogen-free water.

### Human Lung Tissue

Lung tissue samples were obtained from subjects with lung cancer undergoing lobectomy or pneumectomy. Twelve patients with bronchogenic carcinoma, without any other systemic or pulmonary diseases, were enrolled in this study. All subjects were smokers and have had no respiratory tract infection within the last month. None of them was taking immunosuppressants within one month before surgery. In addition, lung tissue samples were obtained from 3 patients with pulmonary sarcoidosis who had undergone diagnostic wedge biopsies and from 3 patients with pulmonary tuberculosis who had undergone upper lobectomy due to destructive tuberculoma. Informed consents were obtained from all subjects. The study was approved by the medical ethics committees of the involved institutions.

### Primary Human Alveolar Epithelial Cells Type II

Samples from macroscopically tumor-free lung tissue were cut from the surgical specimens and used for cell isolation procedure as described previously [19]. In brief, the lung tissue was first sliced and slices were washed three times at 4°C in PBS. The washed slices were incubated in sterile dispase solution at 37°C for 45 min. After dispase digestion the lung tissue slices were cut into small, pipetable pieces, and thoroughly pipetted for several min. Crude tissue and cell suspensions were filtered through nylon gauze with meshes of 100 μm, 50 μm, and 20 μm. The resulting single cell suspension was placed on Ficoll separating solution and centrifuged at 800 × g for 20 min. The AEC-II-enriched cells from the interphase were incubated in 100 mm plastic dishes at 37°C in humidified air containing 5% CO2 for 15, 20 and 30 min with seeding of non-adherent cells on fresh dishes for each time interval to remove adherent cells (alveolar macrophages, monocytes, fibroblasts, and endothelial cells). To remove remaining monocytes/macrophages and lymphocytes, antibodies against CD3 (OKT3, ECACC 86022706) and CD14 (HB-246 ATCC) were added and the antibody-binding cells were removed by anti-mouse IgG coated magnetic beads and Magnetic Activated Cell Sorting (MACS) system (Miltenyi Biotec, Bergisch Gladbach, FRG) as suggested by the supplier. Identity of type II alveolar epithelial cells was confirmed by a modified Papanicolaou staining, their alkaline phosphatase activity, and SP-A mRNA expression in RT-PCR (see below). Cell purity was assessed by immunoperoxidase staining with monoclonal antibodies directed against CD3 and CD14 (Immunotech, Marseille, France) as previously described [20]. Viability of the AEC-II after isolation was > 97% as determined by trypan blue exclusion. After the final step of MACS purification, the AEC-II preparations included in this report were free of CD14+ and CD3+ cells as determined by immunocytochemistry. 98 ± 1.3% of cells were identified as AEC-II by the presence of dark blue inclusions as revealed by modified Papanicolaou staining and 93 ± 2.1% of cells were positive for alkaline phosphatase (data not shown). All RNA samples isolated from these AEC-II preparations contained SP-A mRNA, and CD3 and CD14 mRNA were found in four of twelve samples by RT-PCR (data not shown). In order to avoid false positive results from contaminated cells, these four AEC-II preparations were excluded from further experimental data analysis.

### A549 Cell Line

A549 cells were used as the positive control for CCL2, CXCL9, CXCL10, and CXCL11 mRNA expression and protein production upon stimulation with proinflammatory cytokines. Experiments were performed with cells after 7, 8 and 9 passages after thawing and inoculation in culture. Cells were grown on 75 cm^2 ^tissue culture flask in culture medium (CM) (RPMI1640 medium, 10% heat inactivated FCS, 1% penicillin/streptomycin solution, 1% sodium pyruvate solution and 20 mM HEPES) in a humidified atmosphere containing 5% CO_2 _at 37°C for 5 days. After this culture period, cells were removed from plastic surfaces by treatment with trypsin/EDTA solution (0.05/0.02% in PBS) for 10 min at 37°C, washed twice in PBS and suspended in CM.

### Cell Cultures

Immediately after purification, AEC-II were suspended in CM (1 × 10^6 ^cells/ml) and treated with TNF-α (1 – 10 ng/ml), IFN-γ (10 – 100 U/ml) or IL-1β (10 – 100 U/ml) in collagen R-coated 24-well plates at 37°C, 5% CO_2 _atmosphere. A549 cells were plated at 1 × 10^6 ^/ml in 24-well plates in the same culture condition as for AEC-II and stimulated with TNF-α (1 – 10 ng/ml), IFN-γ (50 – 500 U/ml) or IL-1β (50 – 500 U/ml) in different combinations as indicated in the Results section. At the indicated time, cell-free supernatants were harvested and stored at -70°C, and cell pellets were extracted for total RNA. The cell viability after culture always exceeded 95% in both AEC-II and A549 cells as determined by trypan blue exclusion. For samples of RNA from freshly isolated AEC-II or harvested A549 cells, they were subjected to RNA isolation procedures before cultures, henceforth referred to as non-cultured controls.

### Reverse Transcriptase Polymerase Chain Reaction (RT-PCR)

Total RNA was extracted from cells using TRIzol according to manufacturer's instructions (GIBCO BRL). Equal amounts of total RNA from each sample were primed with oligo dT and reverse-transcribed with SuperScript™ RT for 1 h at 37°C to produce complementary DNA (cDNA). The resulting cDNAs (volume of 2.5 μL) were used for the amplification by PCR of specific targets: CCL2, CXCL10, CXCL11, CXCL9, SPA, and the housekeeping gene β-actin. To demonstrate that RNA samples from AEC-II were not contaminated by RNAs from other types of cells (lymphocytes or alveolar macrophages (AM)) CD3- and CD14-specific primers were also used. All primers were intron-spanning to avoid false positive results by contamination with genomic DNA (Table 1). Target cDNA was amplified using a three-step PCR and an automated thermocycler (Biometra, Göttingen, FRG) according to Murray et al. [21] with primer pairs for CD3 and CD14, and as previously described [19] with primer pairs for β-actin. PCR conditions for CCL2 amplification included: 95°C for 5 min, 95°C for 30 s, 60°C for 30 s, 72°C for 1 min, and 72°C (terminal extension) for 5 min; for CXCL10, CXCL11, and CXCL9: 94°C for 1 min, 53°C for 1 min, 72°C for 2 min; and for SP-A: 94°C for 1 min, 54°C for 1 min, 72°C for 1 min 30 s, and 72°C (terminal extension) for 15 min. The numbers of cycles were the same for all targets (35 cycles), with the exception for SP-A (30 cycles). PCR products (for predicted sizes see Table 1) were electrophoresed on 1.5% agarose gels and stained with GelStar^® ^stain (FMC BioProducts, Rockland, ME). Gel analysis was done densitometrically with "Gel Doc 2000" gel documentation system and "Quantity One 4.0.3" software (Bio-Rad Laboratories, Hercules, CA). To ensure that RNA was effectively reverse transcribed to cDNA for each condition and that stimulation with cytokines by itself did not have any effect on the housekeeping gene β-actin expression, the β-actin PCR was routinely performed in each experiment. To assure the identity of the PCR-amplified fragments, the size of each amplified mRNA fragment was compared with DNA standards (100 bp DNA Ladder; GIBCO BRL, Paisley, Scotland) electrophoresed on the same gel. Additionally, the PCR products were sequenced by the dideoxynucleotide chain-termination method with an autosequencer (ABI PRISM-377, Perkin-Elmer), and their specificity was further confirmed by comparing with the sequence data from the GenBank  database (accession numbers M68519 for SP-A, X14768 for CCL2, AF030514 for CXCL11, NM002416 for CXCL9, and NM001565 for CXCL10) (data not shown). Results are expressed as percent of signal intensities assigned to the target mRNA of the corresponding signal produced by the amplimers for the β-actin gene using the same cDNA specimen.

**Table 1:** Primers used in RT-PCR analysis

cDNA	Primer Sequence*	Product Size (bp)
CCL2	F^† ^: 5'-CAA ACT GAA GCT CGC ACT CTC GCC-3' R^† ^: 5'-ATT CTT GGG TTG TGG AGT GAG TGT TCA-3'	356
CXCL9	F: 5'-CGT GGT AAA ACA CTT GCG GAT ATT-3' R: 5'-CAA TCA TGC TTC CAC TAA CCG ACT-3'	376
CXCL10	F: 5'-CCA TGA ATC AAA CTG CGA TTC TG-3' R: 5'-CTT GGA AGC ACT GCA TCG ATT T-3'	338
CXCL11	F: 5'-AAA GGC TGG TTA CCA TCG GAG T-3' R: 5'-RTGT TGC CAG TAT CCC ATA GCG T-3'	444
CD3	F: 5'-GGC TGT CCT CAT CCT GGC TAT CAT-3' R: 5'-ACT GGT TTC CTT GAA GGT GGC TGT-3'	517
CD14	F: 5'-ACT CCC TCA ATC TGT CGT TCG CTG-3' R: 5'-CTG AAG CCA AGG CAG TTT GAG TCC-3'	341
SP-A	F: 5'-TCT TTG GAT GCC AAC TCA GC-3' R: 5'-CTT TAT TCA GCT CAG GGG TG-3'	666
β-actin	F: 5'-AGC GGG AAA TCG TGC GTG-3' R: 5'-CAG GGT ACA TGG TGG TGCC-3'	309

*All primers were synthesized by MWG-Biotech (MWG-Biotech AG, Ebersberg, FRG); ^† ^F and R denote forward and reverse primer respectively

### Measurement of CCL2 and CXCL10 Concentrations

Chemokines concentrations in A549 cell and primary cultured AEC-II supernatants were measured in duplicate by commercial available ELISA kits. Human CCL2 and CXCL10 ELISA kits were from HyCult biotechnology (Uden, the Netherlands). The assays were performed as suggested by the suppliers. Optical density readings were obtained with a MRX Microplate Reader and analyzed with Revelation 2.0 software (both from Dynex Technologies, FRG). The lower detection limit of the assays was 10 pg/ml for CCL2 and 20 pg/ml for CXCL10. For duplicate samples an intra assay coefficient of variation (CV) of < 10% and interassay CV of < 20% was accepted.

### *In Situ *Hybridization (ISH)

Paraffin embedded lung tissue samples were prepared from the same surgical specimens as described above and used for ISH. These tissue samples showed normal architecture with few intra-alveolar macrophages and edema. Some lung tissue explants were placed in CM alone or with IFN-γ (500 U/ml) and IL-1β (500 U/ml), and/or 10^-4 ^M dexamethasone and incubated at 37°C in humidified air containing 5% CO2 for 24 h. After incubation, these lung tissue explants were further used for ISH. The cDNA probes corresponding to CCL2 and CXCL10 mRNAs were produced by PCR as described before, filtered through Centri-Sep spin columns (Applied Biosystems, Foster City, CA), and labeled with digoxigenin (DIG) following the manufacturer's instructions (Dig-High-Prime, Roche, FRG). After deparaffinization, in situ hybridization was carried out overnight and, after washing at high stringency, detection was performed by application of Anti-Dig/alkaline-phosphatase-conjugate and new-fuchsin as substrate for alkaline phosphatase [22]. Slides were counterstained with Mayers hemalum and mounted with Kayser's glyceringelatine. For negative control, sections were hybridized with hybridization buffer in the absence of labeled cDNA probes. Hybridization of a probe targeting the mRNA of SP-A, a specific product of AEC-II, served as an additional positive control.

### Statistical Analysis

Data are expressed as means ± SEM. Statistical comparisons were made by ANOVA with post hoc Fisher's protected least significant difference (PLSD) for each agent separately. Probability values were considered significant if they were less than 0.05. All testing was done using StatView 5.0 program (SAS Institute Inc., Cary, NC) for Macintosh computers.

## Results

### Chemokine mRNA expression by A549 cells

**Figure 1:**
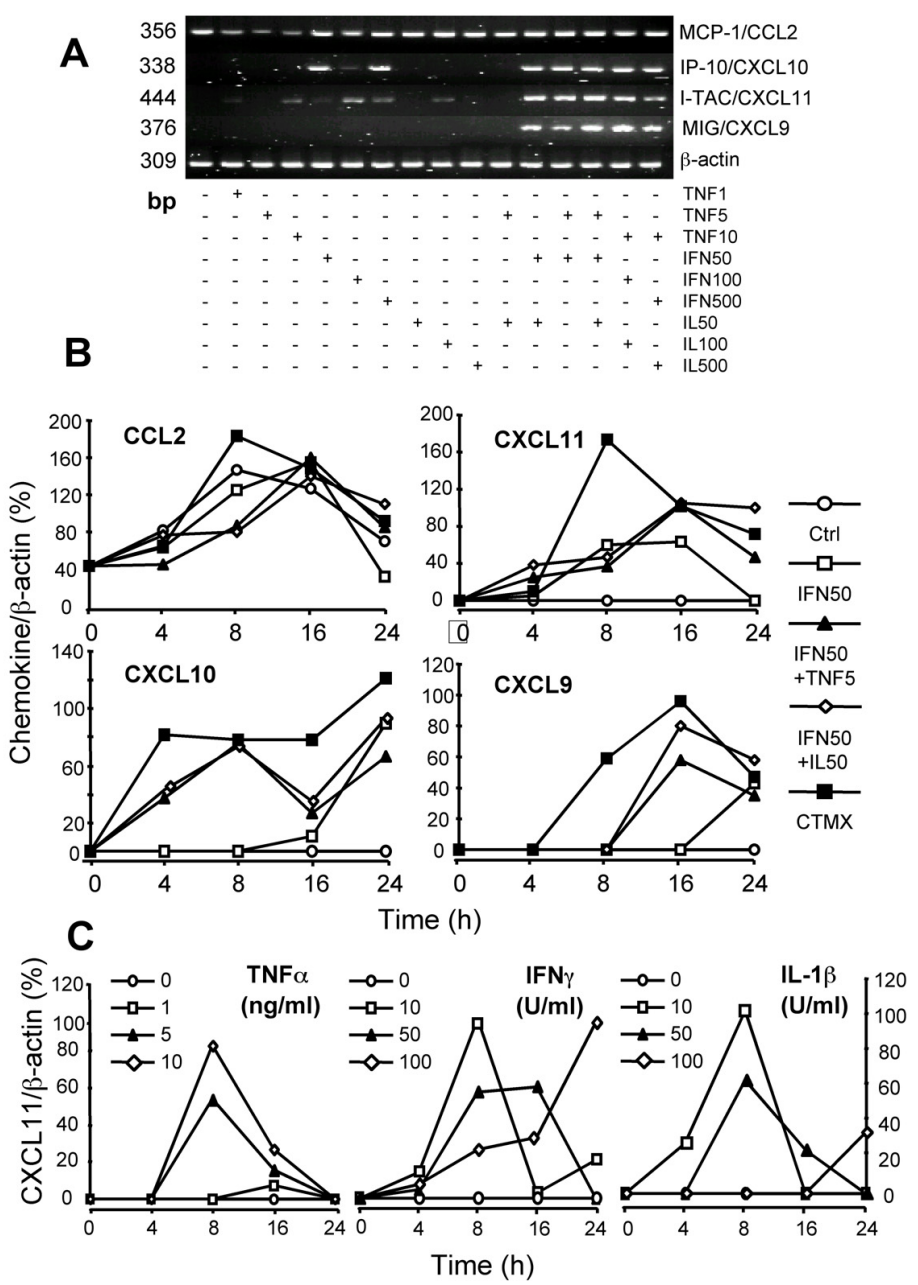
mRNA expression of CCL2 and CXCR3 agonistic chemokines by A549 cells. **A **: Dose response of proinflammatory cytokine-induced CCL2, CXCL10, CXCL11, and CXCL9 mRNA accumulation. The representative gel images from one out of three independent experiments are shown. Expression of β-actin in the same samples demonstrates equal loading of lanes. **B **: Densitometric analysis of the CCL2, CXCL10, CXCL11, and CXCL9 mRNA expression. RT-PCR was performed with total RNA obtained from A549 cells stimulated for the indicated times with 50 U/ml IFN-γ (IFN50), 50 U/ml IFN-γ and 5 ng/ml TNF-α (IFN50+TNF5) or 50 U/ml IL-1β (IFN50+IL50), and a combination of cytokines (CTMX) 50 U/ml of IFN-γ and IL-1β, and 5 ng/ml TNF-α. The mRNA-amplificates from each culture was quantitated individually. The distinct dots on the lines represent the mean percentages of β-actin density of duplicate determinations at each individual time-point for different concentrations/combinations of cytokines. Data are from one representative experiment out of three. **C **: Dose- and time-dependent effects of TNF-α, IFN-γ, and IL-1β at indicated concentrations on CXCL11 mRNA expression by A549 cells are shown.

In preliminary experiments, RT-PCR was performed on the AEC-II-like cell line A549 to assess the spectrum of chemokine mRNA expression at baseline and in response to 24-h stimulation by TNF-α, IFN-γ, and IL-1β at different concentrations. In the same experiments, we also investigated the effects of the combinations of the above mentioned cytokines and different culture periods on chemokine mRNA expression by A549 cells. A549 cells spontaneously expressed mRNA for CCL2 (Figure 1A), and there was a moderate enhancement within 24 h of culture (Figure 1B). Stimulation with TNF-α, IFN-γ or IL-1β resulted in modulation of the steady-state level of CCL2 mRNA within 24 h, and at the end-time point of cultures proinflammatory cytokines slightly increased CCL2 mRNA expression level in a concentration-dependent manner (Figure 1B). Although the differences of CCL2 mRNA accumulation in non-stimulated and TNF-α-, IFN-γ-, or IL-1β-stimulated A549 cells were not obvious, probably due to the high baseline level of CCL2 expression, stimulation with the combination of TNF-α, IFN-γ, and IL-1β led to higher levels of CCL2 mRNA accumulation in a time-dependent fashion (Figure 1B). In contrast, CXCL10, CXCL11, and CXCL9 transcripts were not detected in non-stimulated A549 cells. As shown in Figure 1, resting A549 cells, as well as TNF-α- or IL-1β-treated cells, do not express detectable amounts of CXCL10 or CXCL9 mRNA. Although no detectable amount of CXCL11 transcripts was found in non-stimulated A549 cells, the stimulation with TNF-α, IL-1β or IFN-γ strongly induced CXCL11 mRNA expression (Figure 1A and 1C). IFN-γ alone induced mRNA expression of CXCL10, but not CXCL9, in a dose- and time-dependent manner (Figure 1A and 1B). A considerable accumulation of CXCL10 and CXCL9 mRNA was observed in A549 cells stimulated with IFN-γ plus, either IL-1β or TNF-α, with maximal expression levels being reached by 16 h for CXCL9 and by 24 h for CXCL10, respectively (Figure 1A and 1B). CXCL10, CXCL11, and CXCL9 transcripts were also highly increased by stimulation with combinations of IFN-γ, IL-1β, and TNF-α at different concentrations (Figure 1A and 1B). CXCL11 gene appears to be more sensitive on cytokine mediated induction than CXCL10 and CXCL9. The level of CXCL11 mRNA was increased within 8 h, and declined to baseline at 24 h in the presence of TNF-α or IL-1β in a time- and dose-dependent manner. IFN-γ clearly up-regulated the accumulation of CXCL11 mRNA at all concentrations tested (Figure 1C). Although kinetics of CXCL10, CXCL11, and CXCL9 mRNA expression in IFN-γ-stimulated A549 cells differed greatly from those of IFN-γ plus IL-1β plus TNF-α cells (as in the former conditions, CXCL11 and CXCL10 transcripts reached a maximum at 16 or 24 h, whereas in the latter relatively high levels of chemokine mRNA were detected at 4 or 8 h), it is evident that IFN-γ represents the most potent stimulus to induce mRNA expression of all three CXCR3 agonistic chemokines and that IL-1β and TNF-α exaggerate the up-regulatory effect of IFN-γ in A549 cell line (Figure 1B).

### Chemokine mRNA Expression by AEC-II in Primary Culture

**Figure 2:**
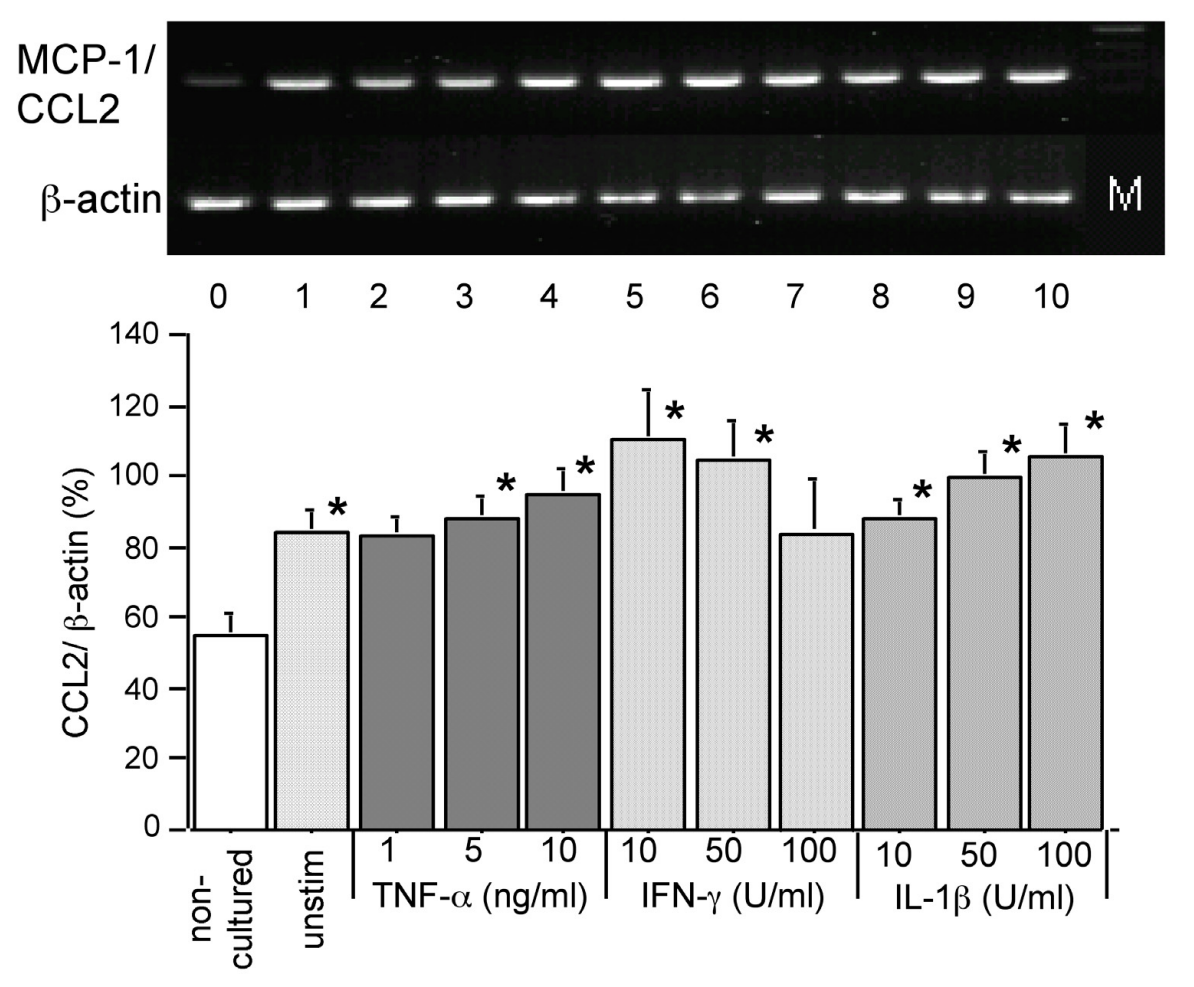
CCL2 mRNA expression by primary cultured AEC-II in response to proinflammatory cytokine stimulation for 24 h. Upper part of figure shows representative images of CCL2 mRNA amplificates in AEC-II derived from one of eight identical experiments. Expression of β-actin in the same samples demonstrates equal loading of lanes. Line 0 – 10 represent cells non-cultured, non-stimulated and stimulated with TNF-α, IFN-γ, or IL-1β, respectively. Line M indicates the molecular weight marker. The lower part of figure shows the results of densitometric analysis of the CCL2 mRNA expression. The mRNA-amplificates from each culture were quantitated individually. Values presented are the mean percentages of β-actin density ± SEM calculated from eight independent experiments. *P < 0.05 compared with non-cultured cells.

Next we examined the effects of proinflammatory cytokines on the expression of chemokine genes expression by human AEC-II to determine whether a similar pattern of mRNA expression and induction as in A549 cells is also detectable in primary AEC-II. As experiments employing A549 cells showed that chemokine mRNA expression levels peaked 24 h after stimulation with proinflammatory cytokines, we used this time point to study the effect of different doses of TNF-α, IFN-γ, and IL-1β on CCL2, CXCL10, CXCL11, and CXCL9 mRNA accumulation in primary cultured AEC-II. We found that non-cultured AEC-II expressed detectable amounts of CCL2 mRNA, which were significantly increased by culture with or without cytokine stimulation (Figure 2, *P *< 0.01, n = 8). TNF-α and IL-1β slightly increased CCL2 mRNA accumulation in a dose-dependent fashion, but this was not statistically significant compared with non-stimulated cells (Figure 2, *P *> 0.05, n = 8). The maximum level of CCL2 mRNA expression was seen in cells stimulated with 10 U/ml of IFN-γ gradually decreasing to baseline values with increasing of IFN-γ concentration (Figure 2).

**Figure 3:**
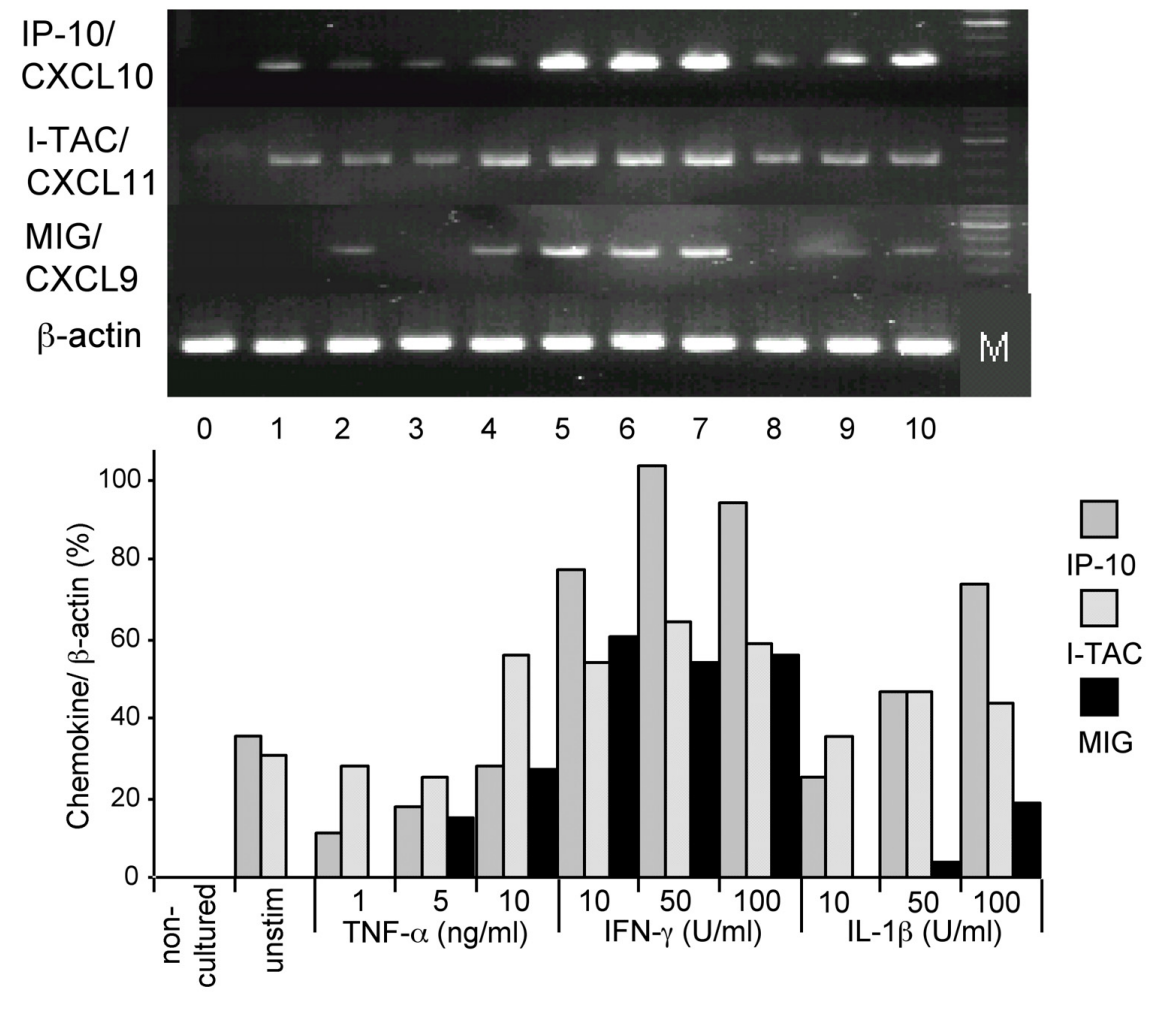
Effect of TNF-α, IFN-γ, and IL-1β stimulation at indicated concentrations on CXCL10, CXCL11, and CXCL9 mRNA expression by primary cultured AEC-II. One representative image of eight independent experiments for each chemokine is shown in the upper part of figure. Expression of β-actin in the same samples demonstrates equal loading of lanes. Line 0 – 10 represent cells non-cultured, non-stimulated and stimulated with TNF-α, IFN-γ or IL-1β respectively. Line M indicates the molecular marker. The lower part of figure shows the results of densitometric analysis of CXCL10, CXCL11, and CXCL9 mRNA expression in AEC-II isolated from one individual. Each panel shows the mean values of duplicate assays for each condition from one experiment representative of eight.

The CXCL mRNA expression pattern of primary AEC-II was similar to that of A549 cells, with some peculiarities in cytokine-stimulated cells. As shown in Figure 3, neither non-cultured nor non-stimulated AEC-II expressed detectable amount of CXCL9 mRNA in all experiments performed. In contrast to A549, CXCL9 mRNA was detected in AEC-II after TNF-α, IL-1β and, more strongly, after IFN-γ treatment. CXCL11 and CXCL10 mRNA were expressed in non-stimulated AEC-II after 4 h of culture, peaked at 16 h and slightly decreased thereafter (Figure 3 and not shown). However, both CXCL10 and CXCL11 were expressed at relatively higher levels after IL-1β, and especially, after IFN-γ treatment in a dose-dependent manner (Figure 3). We did not study the effects of different cytokine combinations on the chemokine mRNA expression patterns due to the strait in amounts of pure human AEC-II isolated from lung tissue samples. No changes in SP-A mRNA expression of non-stimulated or cytokine-stimulated AEC-II were detected after 24 h cultures (data not shown).

### Production of CCL2 and CXCL10 by AEC-II in Primary Culture

**Figure 4:**
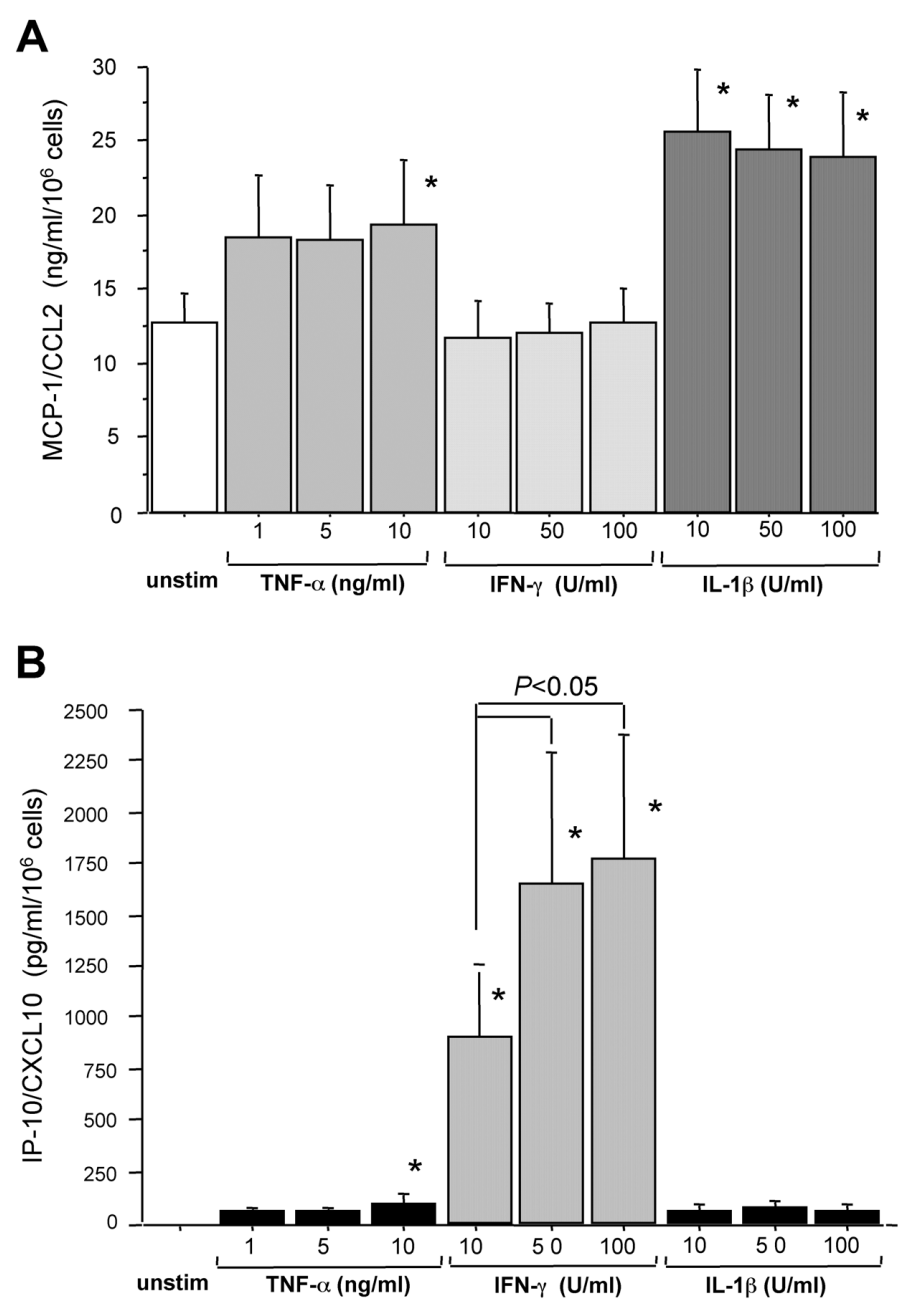
(**A **) CCL2 and (**B **) CXCL10 immunoreactivity in AEC-II supernatants after TNF-α, IFN-γ, and IL-1β stimulation at indicated concentrations for 24 h. Values presented are means ± SEM (n = 12). Statistically significant differences from non-stimulated cells assessed by ANOVA with PLSD separately for each cytokine are indicated by an asterisk (*P < 0.05).

Because CXCL10 mRNA was strongly up-regulated in AEC-II after cytokine stimulation and CCL2 mRNA was detected even in non-stimulated cells, we measured the concentrations of these chemokines in supernatants of AEC-II cultures in the presence or absence of proinflammatory cytokines. In accordance with mRNA expression patterns of CCL2 and CXCL10, AEC-II spontaneously release CCL2 at concentration of 12.7 ± 2.0 ng/ml/10^6 ^cells (Figure 4A), and no detectable amounts of CXCL10 were released by non-stimulated AEC-II after 24 h of cultures (Figure 4B). As shown in Figure 4A, treatment of the AEC-II with IL-1β caused a significant increase in the production of CCL2 (10 U/ml of IL-1β: 25.5 ± 4.2; 50 U/ml: 24.4 ± 3.6; 100 U/ml: 23.8 ± 4.4 ng/mL/10^6 ^cells respectively, *P *< 0.05, n = 12). TNF-α slightly increased the CCL2 release at concentrations of 1 ng/ml (18.4 ± 4.2 ng/ml/10^6 ^cells), 5 ng/ml (18.2 ± 3.8 ng/ml/10^6 ^cells) and 10 ng/ml (19.2 ± 4.4 ng/ml/10^6 ^cells), however, this effect was statistically significant only for a TNF-α concentration of 10 ng/ml (*P *< 0.05; Figure 4A). However, IFN-γ did not significantly change CCL2 protein levels in AEC-II cultures compared with non-stimulated controls (Figure 4A). In marked contrast, AEC-II generated ng/ml quantities of CXCL10 upon stimulation with IFN-γ after 24 h, but TNF-α and IL-1β exerted only marginal effects. As seen in Figure 4B, 10 U/ml of IFN-γ was sufficient to induce a significant increase in the CXCL10 generation by AEC-II being maximal at 100 U/ml of cytokine. A TNF-α concentration of 10 ng/ml slightly, but statistically significantly increased CXCL10 generation by AEC-II compared with non-stimulated controls (Figure 4B).

**Figure 5:**
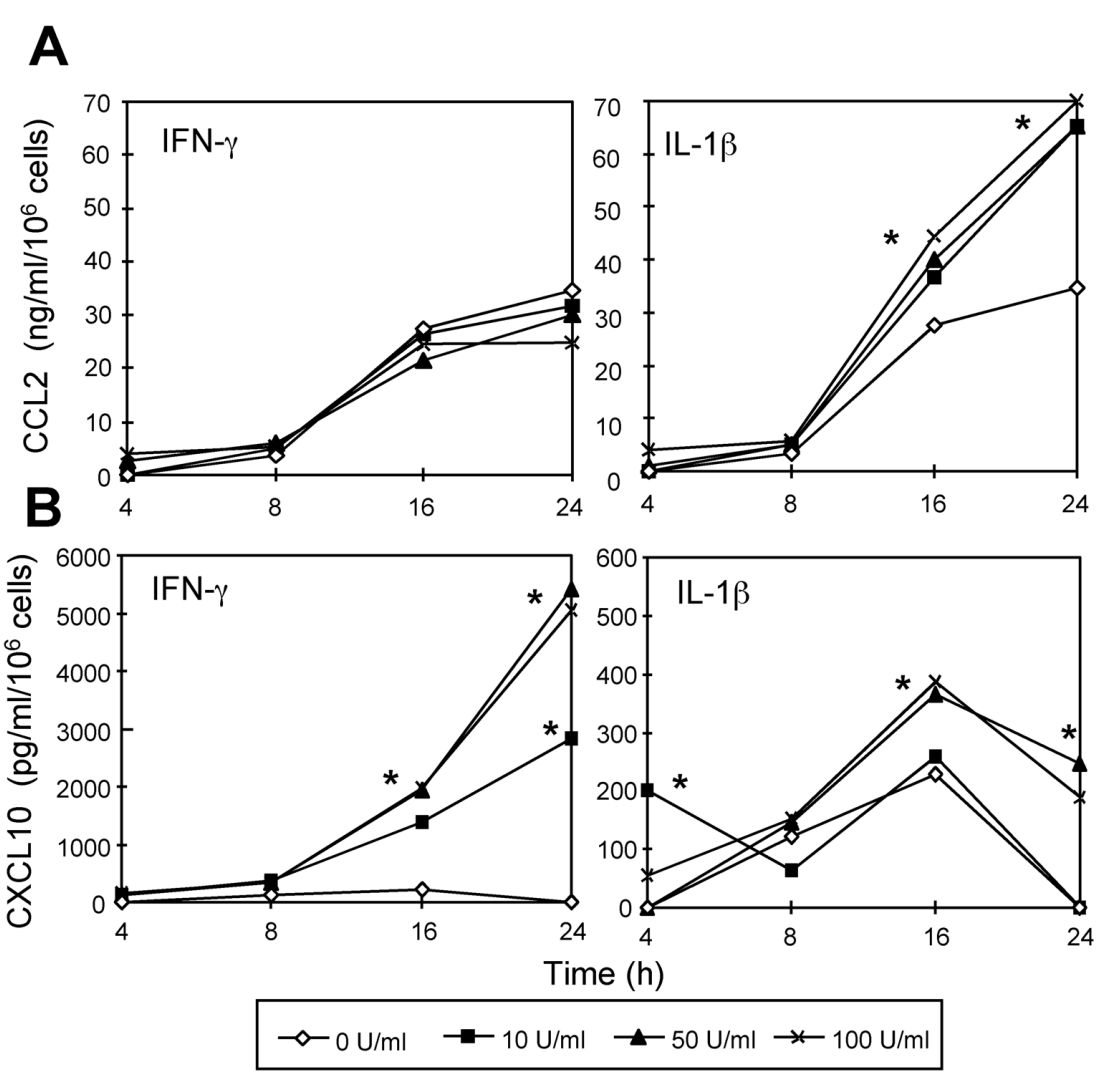
Time course of spontaneous and cytokine-induced (**A **) CCL2 and (**B **) CXCL10 production by primary cultured AEC-II. IL-1β, but not IFN-γ increases CCL2 release, whereas both IL-1β and IFN- increase CXCL10 release in a time- and dose-dependent manner. The increase induced by IL-1β is about 10-fold lower compared with the CXCL10 levels induced by IFN-γ at the same concentrations and time points. The distinct dots on the lines represent means of triplicate determinations at each time-point for different concentrations of cytokine. *P < 0.05 compared to spontaneously produced chemokine levels by AEC-II. Data are representative results from three independent experiments.

Since IL-1β and IFN-γ disclosed the highest differences in the stimulatory capacity for CCL2 and CXCL10 releases in 24-h AEC-II cultures, the effects of both cytokines were analyzed in more detail. As shown in Figure 5A, IL-1β increased CCL2 release in a time- and dose-dependent manner. The IL-1β-induced increase in CCL2 production could be detected as early as 4 h after stimulation and significantly increased with time (Figure 5A). Just within the first 4 h of AEC-II cultures IFN-γ induced a modest CCL2 release, which did not differ statistically significantly from controls. Conversely, with the increase of culture time IFN-γ concentration-dependently decreased CCL2 release of AEC-II in a non-significant magnitude (Figure 5A). The dose- and time-dependent increases of IFN-γ and IL-1β on CXCL10 generation are shown in Figure 5B. A clear-cut dose- and time dependency as seen for stimulation with IFN-γ could also be observed for IL-1β. The low CXCL10 background release increased significantly in the presence of 50 or 100 U/ml IL-1β at time points 16 and 24 h. However, this increase is about 10-fold lower compared with the CXCL10 levels induced by IFN-γ at the same concentrations and time points (Figure 5A and 5B). Experiments with the cell line A549 demonstrated that non-stimulated cells generate significantly lower levels of immunoreactive CCL2 (*P *< 0.01; 2.3 ± 0.9 ng/ml/10^6 ^cells after 24 h, n = 6) compared with primary cultured AEC-II (data not shown). Additionally, TNF-α, but not IFN-γ or IL-1β, up-regulated CCL2 release, and this effect was only seen after 4 h of culture (data not shown). A549 cells also released CXCL10, and consistent with the mRNA data, a combination of IFN-γ with IL-1β and/or TNF-α significantly up-regulated CXCL10 release by these cells. IFN-γ/IL-1β/TNF-α-stimulated A549 cells generated 5.3 ± 1.9 ng/ml/10^6 ^cells (n = 3) of CXCL10 protein for 24 h, which was a 50-fold increase over IFN-γ-stimulated cells (data not shown).

### CCL2 and CXCL10 mRNA expression by AEC-II *in vivo *

**Figure 6:**
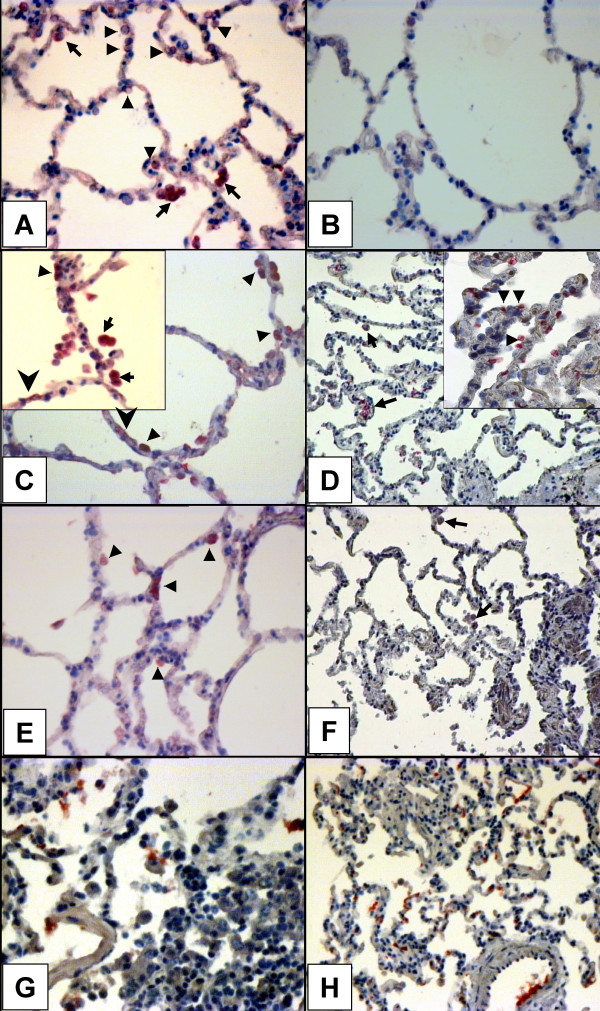
Localization of CCL2 and CXCL10 mRNA in lung tissue explants non-cultured and cultured with IL-1β and IFN-γ, in the presence or absence of 10^-4 ^M dexamethasone (DEX) for 24 h. CCL2 mRNA is constitutively produced in normal lung tissue by AEC-II (arrowheads) and AM (arrows) (**A **), and stimulation with 500 U/ml of human recombinant IL-1β and IFN-γ (**C **, inset) significantly up-regulates CCL2 mRNA expression by these cells compared to non-cultured (**A **) or 24-h cultured controls (data not shown). AEC-I also show weak positive signal for CCL2 mRNA (**C **, sharp arrowheads). DEX treatment leads to inhibition of the IL-1β-induced CCL2 mRNA production in AEC-II (**E **and **C **), but does not change the basal expression (**E **and **A **). Panels **B **, **D **, and **F **show the same lung tissue explants treated as indicated above and hybridized with a CXCL10 specific probe. Panel **D **(inset) illustrates a prominent inducible effect of cytokine-stimulation on CXCL10 mRNA expression *in situ *by AEC-II (arrowheads) and AM (arrows), and panel **F **depicts the inhibitory effect of DEX on IFN-γ/IL-1β-induced CXCL10 mRNA expression. Data of one representative experiment from five are shown. Panels **G **and **H **illustrate CXCL10 mRNA localization in the lung from patients with pulmonary sarcoidosis and tuberculosis respectively. Control slides, in which hybridization buffer alone was applied, display no reactivity in all experiments (data not shown).

To determine if AEC-II expression of those chemokines can also be regulated *in vivo *, we took advantage of an *in situ *hybridization (ISH) method. ISH using DIG-labeled cDNA probes detected specific signals for CCL2 mRNA mainly in intra-alveolar macrophages in all lung tissue preparations included in the present study. Positive signals for CCL2 mRNA were also detected in AEC-II, which were typically localized at alveolar corners and exhibited cuboidal morphology (Figure 6A, arrowheads). After treatment with IL-1β almost all AEC-II displayed strong positive signal for CCL2 mRNA (Figure 6C, arrowheads). The same pattern of CCL2 mRNA expression was observed in both macrophages located in the alveolar lumen and those adjacent to alveolar epithelium (Figure 6C, inset, arrows). Interestingly, a weak positive signal was also detected in AEC type I (Figure 6C, sharp arrowheads). Dexamethasone treatment markedly inhibited IL-1β-induced CCL2 expression, but did not change basal levels compared to non-stimulated (data not shown) or non-cultured samples (Figure 6E and 6A). In contrast to CCL2, no positive signals for CXCL10 mRNA were detected in tissue explants from normal lungs (Figure 6B). However, after stimulation of whole lung tissue explants for 24 h *in vitro *with IL-1β and IFN-γ, in AEC-II (Figure 6D, inset, arrowheads) as well as in AM clear positive signals for CXCL10 mRNA could be detected (Figure 6D, arrows). Treatment with dexamethasone almost completely suppressed cytokine-induced CXCL10 mRNA in AEC-II and AM (Figure 6F, arrows). In situ hybridization was also performed on lung tissue preparations obtained from patients with pulmonary sarcoidosis and tuberculosis. The strong positive signals of CXCL10 mRNA were observed in AM and AEC-II on the perifocal zones of sarcoid granulomas (Figure 6G) and in the alveolar epithelium on tuberculous lung tissue preparations (Figure 6H). The specific signals were not detected in control preparations, in which specific DNA probes were substituted by hybridization buffer (not shown). For control purposes SP-A mRNA predominantly localized in AEC-II was detected in all lung tissue preparations (data not shown).

## Discussion

To increase our knowledge in mechanisms controlling the recruitment and activation of inflammatory cells in the alveolar space and the role of alveolar epithelial cells type II in the cytokine network of the lung, we investigated the effects of proinflammatory cytokines on chemokine gene expression and production by human primary AEC-II. We examined CCL2, a CC chemokine that attracts predominantly monocytes/macrophages and activated T cells by binding to CCR2, and CXCL9, CXCL10, and CXCL11, T cell-specific chemokines binding to CXCR3. In this work, we demonstrate that CCL2 mRNA is present in freshly isolated AEC-II and its level is significantly up-regulated during culture. The proinflammatory cytokines IL-1β, TNF-α, and IFN-γ increased the accumulation of CCL2 mRNA in 24 h cultured AEC-II in a dose-dependent manner, however these effects were not statistically significant compared with non-stimulated cells. CCL2 mRNA patterns of resting and cytokine-stimulated A549 cells, which were used as control, disclosed the same expression profiles as observed in primary AEC-II. The highest CCL2 mRNA level was detected in A549 cells stimulated with a combination of IL-1β, TNF-α, and IFN-γ, and this effect was time-dependent.

In agreement with mRNA data, we also found that significant amounts of CCL2 protein were spontaneously secreted from primary cultured AEC-II, and IL-1β or TNF-α, but not IFN-γ, up-regulated CCL2 production, confirming a previous study [8]. In contrast to studies of IFN-γ effects on CCL2 release by human bronchial and endothelial cells and fibroblasts we could not demonstrate that IFN-γ up-regulates the CCL2 protein production by AEC-II and A549 cells [23-25]. Although, IFN-γ modulates CCL2 mRNA expression in AEC-II, time course experiments showed that IFN-γ does not significantly influence CCL2 release. These results are in line with other observations of IFN-γ being rather an inhibitor than promoter of spontaneous and LPS-induced tissue-specific CCL2 releases [26,27]. Interestingly, IFN-γ also selectively inhibits the CCR2 expression on human monocytes [28]. It seems that differences in IFN-γ regulation of CCL2 may result from differential responses of target cells to pro- and anti-inflammatory stimuli and to cell type-specific patterns of stimulus sensitivity. In contrast to IFN-γ, IL-1β was the most potent stimulus on CCL2 release by AEC-II. More than 25 ng/ml/10^6 ^cells of CCL2 were detected in supernatants collected from AEC-II, activated with IL-1β for 24 h. This is the highest level reported to date for cytokine-stimulated CCL2 secretion from airway epithelial cells. In comparison, in a 24-h period, human bronchial epithelial cells treated with IL-1β released 25-fold less CCL2 [23], and in our experiments, A549 cells maximally stimulated with TNF-α secreted 10-fold less protein. Furthermore, Sadek and associates demonstrated that human AM retrieved from BAL generate the same levels of CCL2 after 72-h stimulation with LPS in culture [29]. Thus, spontaneous CCL2 level produced by AEC-II is of biological importance since on a per cell basis it is three-fold higher than baseline CCL2 level generated by human AM for 72 h *in vitro *[29]. Although we did not determine the capacity of IL-1β-stimulated AEC-II to attract mononuclear cells, previous studies have shown that AEC-II-derived CCL2 is strongly chemotactic for CD14^+ ^and CD3^+ ^cells *in vitro *and *in vivo *[8,10,15].

Under normal condition the alveolar space contains a low number of leukocytes with AM forming about 95% of total cell population. As AEC-II are uniquely positioned in the borders between the microvascular compartment and the alveolar space, and constitutively generate considerable amounts of CCL2, one may hypothesize that this AEC-II-derived chemokine is responsible, at least partially, for basal recruitment of monocytes and their differentiation into AM, in healthy humans. In this respect Gunn and colleagues have demonstrated that CCL2 overexpression in the lung of transgenic mice leads to a marked increase in the number of BAL monocytes, but does not cause inflammatory activation of cells [15]. According with our *in vitro *and *in vivo *results, it is necessary to have additional inflammatory agonists such as macrophage-derived IL-1β and/or TNF-α for increasing and amplifying CCL2 expression by AEC-II, which in turn might be important factors for further development and manifestation of lung inflammation.

In contrast to CCL2, we could demonstrate that IFN-γ induces the expression of CXCL9, CXCL10, and CXCL11 by AEC-II. In fact, IFN-γ significantly up-regulated CXCL9, CXCL10, and CXCL11 mRNA accumulation and CXCL10 production of AEC-II and A549 cells. Albeit at low levels, CXCL10, CXCL11, and CXCL9 were also induced directly by IL-1β and TNF-α in primary cultured AEC-II. Interestingly, the kinetics of CXCL11 mRNA expression in IFN-γ- or IFN-γ plus TNF-α and IL-1β-treated A549 cells were faster than those of CXCL9 or CXCL10 mRNA expression. Furthermore, the effect of TNF-α and IL-1β was more pronounced on CXCL11 mRNA accumulation in A549 cells compared with CXCL10 or CXCL9, suggesting that different pathways might be involved in CXCL9 and CXCL10 expression at one side, and CXCL11 on the other side, even though they are all induced by IFN-γ. The significance of a sequential regulated expression of CXCL10, CXCL11, and CXCL9 by AEC-II is only a matter of speculation, but a similar pattern has been found in other cell types, for instance, in endothelial cell, bronchial epithelial cells, and neutrophils [30-32]. CXCL10 mRNA accumulation in primary cultured AEC-II and in the control A549 cells closely reflected levels of secreted CXCL10 protein, as previously shown in human bronchial epithelial cells [31], suggesting that the CXCL10 production is strongly regulated and dependent on the transcriptional mechanisms.

To date, a number of cellular sources of CCL2 and CXCL10 in the inflamed lung have been identified, including macrophages, fibroblasts, airway epithelium, and endothelial cells. In the present study, we have evaluated the ability of human AEC-II to express and produce CCL2 and CXCL10 *in vitro *, and express CCL2 and CXCL10 mRNA *in situ *. ISH studies revealed intense positive signals for CXCL10 mRNA in AEC-II, as well as in interstitial and alveolar macrophages in lung tissue explants after *in vitro *treatment with IL-1β and/or IFN-γ. In contrast, no positive signal was detected in non-cultured or cultured lung tissue explants with medium alone or with IFN-γ/IL-1β and 10^-4 ^M dexamethasone. On the other hand, CCL2 mRNA expression was detected in AEC-II and AM in all lung tissue explants, and IL-1β, alone or in combination with IFN-γ, markedly up-regulated CCL2 mRNA expression of AEC-II and AM *in situ *. Treatment with dexamethasone attenuated the signal intensities in cytokine-stimulated, but not in non-stimulated preparations. These results from *in vitro *stimulation and dexamethasone inhibition experiments demonstrated that AEC-II were capable of expressing significant quantities of CXCL10 mRNA *in situ *only under local IFN-γ or IL-1β activation, in contrast to CCL2, which is expressed constitutively, and proinflammatory cytokines only up-modulated the steady state mRNA levels of CCL2. These data corroborate well observations from van der Velden et al. and Witowski et al., which have shown that dexamethasone and actinomycin D strongly inhibit cytokine-driven but not constitutive CCL2 release in human bronchial epithelium and peritoneal fibroblasts [23,33].

Our *in situ *hybridization data are consistent with observations from Jaffe and associates, and Martin et al., which have shown that only after local, but not after systemic administration of recombinant human IFN-γ in healthy volunteers, CXCL10 mRNA was induced in AM [34,35]. Moreover, production of CXCL10 and CXCL9 in the injured lung was not completely suppressed in mice deficient for IFN-γ or the IFN-γ receptor [36], and CXCL10, but not CCL2, failed in the human T cell transendothelial migration model [37]. All these data suggest that in contrast to CCL2, CXCL10 is directly induced by IFN-γ released from cells within the lung rather than by IFN-γ derived from distant sites, and that alternate agonists are present in the alveolar compartment, which may together with IFN-γ or separately amplify CXCL10 expression, and subsequently promote local accumulation of CXCR3^+ ^cells in the alveolar space. In addition, ISH data strongly suggest that effects of the proinflammatory cytokines on CCL2 and CXCL10 mRNA accumulation and protein generation in primary cultured AEC-II were not due to artifacts elicited by cell isolation procedure or culture conditions.

The quantitative differences, time and cytokine-inducing profile dependencies in CCL2, CXCL9, CXCL10, and CXCL11 expression of AEC-II suggest that there are several cytokine/chemokine cascades within the injured lung, which in turn, determine the flexible programs of recruitment and activation of inflammatory cells into the alveolar space. For instance, alveolar macrophage-derived pro-inflammatory cytokines such as IL-1 and TNF-α, but not IFN-γ, directly up-regulate expression by AEC-II of certain chemokines including CCL2 and CXCL8 [8,10,19,38]. In our previous report we demonstrated that in close similarity with CCL2, TNF-α and IL-1β, but not IFN-γ, up-modulated constitutive interleukin-8/IL-8 (CXCL8) release in primary cultured AEC-II, and cytokine-derived increase of CXCL8 basal level was only two-fold lower than LPS-induced CXCL8 release in AM [19]. Our results demonstrating high levels of CCL2, CXCL8 and CXCL10 production by AEC-II *in vitro *indicate that AEC-II have the potential to participate in physiologic and pathologic macrophage, neutrophil and T cell responses within the alveolar space. By chemoattracting IL-1-producing monocytes/macrophages, TNF-α-producing neutrophils and IFN-γ-producing CD4^+ ^or CD8^+ ^cells in close proximity to the epithelium, AEC-II-derived CCL2, CXCL8, CXCL9, CXCL10, and CXCL11, which themselves are up-regulated by IL-1β and/or TNF-α, and IFN-γ, may activate several positive feedback loops. In addition, it has been reported that CXCL10 selectively activated and enhanced antigen-driven IFN-γ gene expression in T cells [39]. Thus, it is tempting to speculate that locally produced CCL2 and CXCL10 by AEC-II attract activated memory T cells into alveoli and further amplify antigen-driven IFN-γ response of Th1 cells. The strong confirmation of this hypothesis results from animal models of the lung inflammation and some clinical observations in pulmonary diseases. It has been demonstrated that bronchoalveolar lavage (BAL) cells disclosed the Th1-dominated pattern in transgenic rats that express CXCL10 in the lung, and BAL lymphocytes of HIV-infected patients with T-cell alveolitis were CD8+ T cells expressing high levels of CXCR3 and IFN-γ, which exhibited a high migratory capability in response to CXCL10 and CXCL9 [40,41]. Similarly, over-expression of human CCL2 in transgenic AEC-II or intra-tracheal administration of murine MCP-1 caused a substantial accumulation of activated monocytes within the bronchoalveolar space of mice [15,16]. Additionally, Miotto et al. reported that CXCL10 expression was strongly up-regulated in Th1-mediated lung diseases, whereas increased CCL2 expression was not specifically associated with Th1 or Th2 patterns[3]. Our *in situ *hybridization data confirm that in contrast with normal lung tissue preparations a strong expression of CXCL10 can be detected in sarcoidosis and tuberculosis – diseases in which the Th1-type cytokine IFN-γ is up-regulated.

Beside the pro-inflammatory effects of CXCL9, CXCL10, and CXCL11 they also exhibit down-regulation e.g. on the migration of eosinophils [42] or on angiogenesis [43]. This is of interest, because it could be shown in a mouse model that CXCL11 attenuates fibrosis by inhibition of vascular remodeling [44]. In addition, it could be demonstrated that CXCL9 down-regulates IL-4 expression but up-regulates IFNγ expression by T cells. This illustrates that AEC-II not only induce the migration of Th1 cells by the release of CXCR3-ligands but they also participate in T cell activation [20] and Th1 polarization of lung T cells.

Since it is known that AEC-II also express CXCR3A and CXCR3B [45] it is tempting to speculate about a possible activity of CXCL9, CXCL10, and CXCL11 on AEC-II in an autocrine fashion, e.g. in re-epithelialization after lung injury. Therefore, induction of CXCR3 agonists may in some respects of benefit and may represent a possible therapeutic mechanism of IFNγ therapy of IPF patients [46].

## Conclusion

**Figure 7:**
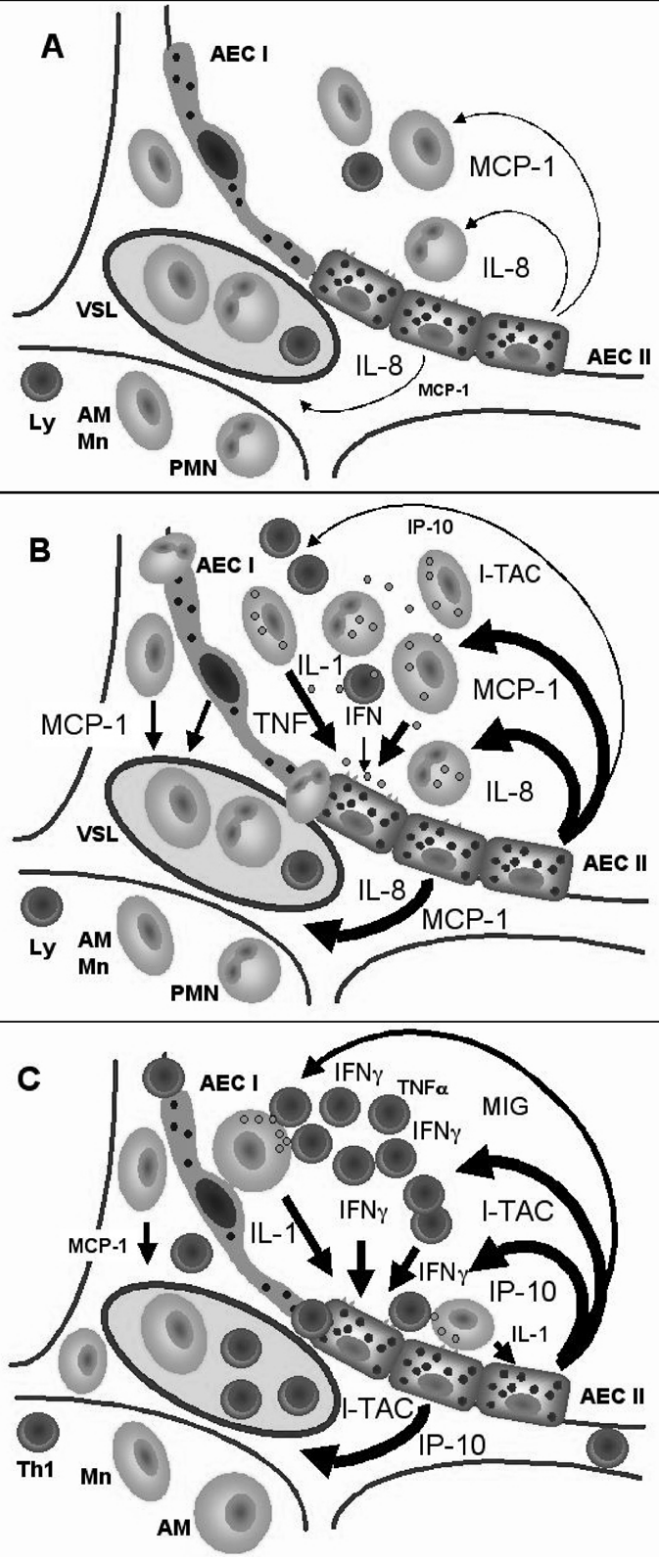
Alveolar epithelial cells type II drive a leukocyte trafficking in host defense and specific immune response. The schematic drawing shows spatial distribution of chemokine expression and leukocyte recruitment: in healthy condition (**A **) two chemokines, MCP-1/CCL2 and IL-8/CXCL8, are expressed constitutively by AEC-II and provided the physiological influx of monocytes (Mn), neutrophils (PMN), and lymphocytes (Ly) into alveolar space from pulmonary vasculature (VSL); in the initial phase of alveolar inflammation caused by inflammatory agents (**B **) increasing alveolar macrophage (AM) and particularly epithelial expression of CCL2 and CXCL8 as well as the expression of CXCL10 and CXCL11 is paralleled by pronounced recruitment of Mn, PMN, and Ly to the alveolar compartment; further development of the immune response (**C **) leads to the expression of high levels of CXCL9, CXCL10, and CXCL11 in AEC-II induced by IFN-γ and/or TNF-α, and IL-1β and associated with recruitment of activated T cells (Th1) to the interstitium and alveolar space. Mn and PMN reside in the interstitium and vascular compartment, which may be because of an IFN-γ inhibition of CCL2 and CXCL8 expression by AEC-II.

Taken together our data indicate that although AEC-II constitute about 15% of all lung cells, they may play a prominent role in the pathogenesis of inflammatory lung diseases. This study suggests that in human AEC-II, CXCR3 agonistic chemokines are expressed and regulated differently from CCL2. This pattern of differential expression and regulation was also seen when we explored the effect proinflammatory cytokines on the IL-8/CXCL8 expression by human AEC-II [19]. We proposed that CXCL8 and CCL2 constitutively produced by AEC-II may mediate the „steady state" recruitment of blood cells, i.e. monocytes/macrophages and neutrophils, which provide a first line of the host defense in the lung (Figure 7A). Bacteria, endotoxin, viruses, and other pathogens may enhance the production of CXCL8 and CCL2 by AEC-II directly or with monocyte/macrophage derived TNF-α and IL-1β as an intermediary. Cytokine-mediated increases of CCL2 or CXCL8 levels may therefore be considered as an alert signal for leukocytes in the pulmonary vascular bed and alveolar space (Figure 7B). The increased numbers of leukocytes, including activated T cells, and the presence of specific antigen can further elicit the development of specific immune response within alveoli. Subsequently, activated T cells (Th1) released high amounts of IFN-γ resulting in additional CXCL9, CXCL10, or CXCL11 induction by resident cells (AEC-II) of the alveoli. This positive feedback loop establishes a new chemotactic gradient inducing an increased migration of CXCR3^+ ^T cells from the blood stream through the endothelium into both the interstitium and alveolar space (Figure 7C). Under certain pathologic circumstances, several auto-regulatory loops involving alveolar epithelium might be exaggerated, and may contribute to acute and chronic inflammatory injuries of the lung.

## List of abbreviations

AEC-II – alveolar epithelial cells type II, BAL – bronchoalveolar lavage, CCL2 – CC-ligand 2 (= MCP-1 (monocyte chemoattractant protein-1)), CXCL9 – CXC-ligand 9 (= MIG (monokine induced by interferon-γ)), CXCL10 – CXC-ligand 10 (= IP-10 (Interferon-inducible protein-10)), CXCL11 CXC-ligand 11 (= I-TAC (Interferon-inducible T-cell alpha chemoattractant)), IFN-γ – interferon-γ, IL-1β-interleukin-1β, ISH – *in situ *hybridization, SPA – surfactant protein A, TNF-α – tumor necrosis factor α,

## Authors' contributions

DVP isolated the cells, performed the cell culture and PCR, developed specific vectors for ISH and drafted the manuscript. TG performed the ISH, EV conducted the pathological part of the study, CL, AP JMQ performed the clinical part and were involved in the coordination of the study. GZ conceived the study, carried out the ELISAs and was involved in preparing the manuscript.
